# Next-Generation Digital Histopathology of the Tumor Microenvironment

**DOI:** 10.3390/genes12040538

**Published:** 2021-04-07

**Authors:** Felicitas Mungenast, Achala Fernando, Robert Nica, Bogdan Boghiu, Bianca Lungu, Jyotsna Batra, Rupert C. Ecker

**Affiliations:** 1 Institute of Pathophysiology and Allergy Research, Center for Pathophysiology, Infectiology and Immunology, Medical University of Vienna, 1090 Vienna, Austria; 2 TissueGnostics GmbH, 1020 Vienna, Austria; robert.nica@tissuegnostics.com; 3 Translational Research Institute, 37 Kent Street, Woolloongabba, QLD 4102, Australia; achala.vitharanage@hdr.qut.edu.au (A.F.); jyotsna.batra@qut.edu.au (J.B.); 4 School of Biomedical Sciences, Faculty of Health, Queensland University of Technology, Brisbane, QLD 4059, Australia; 5 TissueGnostics SRL, 700028 Iasi, Romania; bogdan.boghiu@tissuegnostics.com (B.B.); bianca.plosnita@tissuegnostics.com (B.L.)

**Keywords:** next-generation digital histopathology, tissue cytometry, multiplexing, RNA ISH, cancer, tumor immune microenvironment, tumor microenvironment

## Abstract

Progress in cancer research is substantially dependent on innovative technologies that permit a concerted analysis of the tumor microenvironment and the cellular phenotypes resulting from somatic mutations and post-translational modifications. In view of a large number of genes, multiplied by differential splicing as well as post-translational protein modifications, the ability to identify and quantify the actual phenotypes of individual cell populations in situ, i.e., in their tissue environment, has become a prerequisite for understanding tumorigenesis and cancer progression. The need for quantitative analyses has led to a renaissance of optical instruments and imaging techniques. With the emergence of precision medicine, automated analysis of a constantly increasing number of cellular markers and their measurement in spatial context have become increasingly necessary to understand the molecular mechanisms that lead to different pathways of disease progression in individual patients. In this review, we summarize the joint effort that academia and industry have undertaken to establish methods and protocols for molecular profiling and immunophenotyping of cancer tissues for next-generation digital histopathology—which is characterized by the use of whole-slide imaging (brightfield, widefield fluorescence, confocal, multispectral, and/or multiplexing technologies) combined with state-of-the-art image cytometry and advanced methods for machine and deep learning.

## 1. Introduction

Cancer is a crucial global health challenge. The incidence of new cancer cases is predicted to increase by around 70% over the coming two decades [1]. Due to the idea that cancer originates from a deranged genome, exploring the genomic, transcriptomic, and proteomic nature of cancer is vital for understanding and utilizing remedies for the treatment of cancer [2]. The tumor cells and the surrounding microenvironment, which includes various types of immune cells, signaling cells and molecules, fibroblasts, and the extracellular matrix comprised of adjacent blood vessels, are highly interdependent compartments. We have started to understand the complex interplay between each of these compartments, with the tumor itself shaping and directing its surroundings—the tumor microenvironment (TME)—while at the same time obtaining signals from this microenvironment for further progression [3]. It is often believed that cancer seeds are germinated primarily in an appropriate microenvironment [4]. Precise localization of molecular indicators by spatial immunophenotyping techniques inside the microenvironment concedes an additional comprehensive analysis of the tumor to foresee its progression and therapy response [5,6,7].

In the past few years, in-depth profiling of cancer cells/tissues has determined the cancer genome, the transcriptome, and the proteome as powerful sources of diagnostic, prognostic, and predictive markers/biomarkers [8,9]. In this regard, spatially mapped cellular gene expression has appeared as a critical method to understand the localization and complicated multicellular interactions of DNA, RNA, and proteins within cells located in the tumor as well as in the TME [10,11]. Interrogation of the tumor cellular organization context at single cell level with the cell’s interactions with neighbor cells helps towards a better understanding of the heterogeneity of the TME between individuals as well as within the same tumor sample [12,13]. Thus, a need arises for multi-omics approaches where many DNA, RNA, splice variants and protein targets can be visualized by various staining techniques in situ. This dictates the need to quantify stained tissue sections, in terms of intensity, presence (expression levels), and/or spatial distribution in an unbiased, objective, fast, and automated way. Next-generation digital pathology is able to fulfil these requirements in research as well as in clinics. Even though the term “digital pathology” has been used for decades, its practical definition is still limited to digitizing samples. The actual analysis in digital pathology is still performed visually—by pathologists looking on a monitor rather than through a microscope’s oculars. Converting immunohistochemistry (IHC), immunofluorescence (IF), or RNA in situ hybridization (RNA ISH) stained markers within tissues sections into digitized images is a prerequisite, but for pathology to become really “digital” and automated, further processing and extraction of quantitative data, termed as image cytometry, is required. Several commercial systems are available [14] that offer specialized software solutions utilizing image cytometry, but are methodically focused on the analysis of histological sections and are thus referred to as tissue cytometry [15,16]. Due to the constant evolvement and the increasing reliability of these systems, two commercially available whole slide imagers are approved by the ‘U.S. Food and Drug Administration’ (FDA) and can be used for clinical approaches [17]. To evaluate the reliability of these next-generation digital pathology platforms in clinics in terms of prognostication and patient management, Nagpal et al. conducted a comprehensive study using prostatectomy specimens. They established a deep convolutional neural network addressing Gleason scoring, which was trained by pathologists on 912 hematoxylin and eosin (HE) stained tissue slides. Next, they compared the classification of 29 additional pathologists with the results of the deep learning-based system. As an outcome of the study, the deep learning-based Gleason classification system showed a significantly higher sensitivity and specificity than 9 out of 10 pathologists [18]. Further studies that used deep convolutional/deep learning/machine learning networks for cancer tissue classification/TME on HE cancer samples for follow-up alignment with clinicopathological parameters are those by Jiao et al. [19], Kwak et al. [20], and Bidal et al. [21] on colon cancer samples, Mittal et al. on breast cancer [22], Wang et al. on lung adenocarcinoma [23], and Diao et al. on skin cutaneous melanoma, stomach adenocarcinoma, breast cancer, lung adenocarcinoma, and lung squamous cell carcinoma [24]. 

All the above-mentioned studies are good examples that show that tissue cytometry may provide the methodological basis for next-generation digital pathology, which is the state-of-the-art technology to use and constitutes an enabling factor for precision medicine in clinics as well as in research. Within this review, we are going one step further by addressing the concepts of next-generation digital pathology using imaging-based tissue cytometry, in combination with multiplexing and RNA ISH technologies, as an emerging and central method within precision diagnostics, and discussing various applications.

## 2. Multiplexing Techniques as Useful Tools for High-Content Phenotyping

To achieve high-content phenotyping, optionally in combination with applying genetic markers for well-defined DNA loci as well as total RNA or specific mRNA measurements, the importance of multiplexing staining techniques continues to increase in research and clinics, especially for the purpose of determining the complex immune and tumor microenvironment status in patients suffering from cancer, graft versus host disease, and other pathological conditions related to immune responses [25]. In clinics the assessment of various immune cell markers as well as immune cell populations is required for prognosis, diagnosis, and selecting the therapeutic intervention strategy. Conventional IHC/IF staining techniques are restricted by the number of markers which can be detected at once within one tissue section. This problem was bypassed by staining consecutive tissue sections, with the main limitation being that high-dimensional co-expression analysis is not possible and very precious information is lost [14]. However, in recent years the ability of multiplexing, in terms of visualizing a high number of markers at one time within a sample, has evolved and thereby represents a powerful tool for investigating complex molecular/functional processes and interactions within cells as well as in the complex native tissue environment. In this section, we discuss various immunohistochemistry and immunofluorescence multiplexing techniques.

IHC-based multiplexing methods: Conventional IHC staining usually used in pathology only allows the detection of one marker per tissue section, and therefore no co-expression analysis is possible. With IHC multiplexing techniques the number of stained markers per tissue sections can be increased drastically, which leads to more detailed staining of patient tissue, especially important for clinical applications in respect to diagnostics and prognosis [26]. Previously published multiplexing methods based on IHC are “multiplexed immunohistochemical consecutive staining on single slide” (MICSSS) [27] and “Sequential Immunoperoxidase Labelling and Erasing Method” (SIMPLE) [28]. These two techniques use the chemical property alcohol solubility of the peroxidase substrate 3-amino-9-ethylcarbazole (AEC). The protocol is similar to conventional IHC but includes after image acquisition the removal of AEC with organic solvent-based destaining buffer, and the restaining with new antibodies targeting other markers of interest. Thereby, MICSSS and SIMPLE enable multiple staining rounds. As a final step, the images taken after each staining round are overlaid and sometimes even transferred into a pseudo-color IF-like image. The advantages of these two staining techniques are that they allow co-expression analysis and there are no limitations in terms of antibody species (same antibody origin species can be used for each marker), which is a limitation in conventional staining IHC techniques. However, MICSSS and SIMPLE allow only one marker at each staining round and therefore are limited in number of markers (accordingly to published data, up to 5–10 in total) and are highly time intensive [28,29].

IF-based multiplexing methods: Multiplexing methods based on immunofluorescence are much more common and comprise many advantages over IHC-based multiplexing methods. With IF multiplexing techniques, conventional immunofluorescence staining/imaging can be extended from around 6 to up to 60 markers. Published IF multiplexing techniques include “TSA Opal multiplex immunohistochemistry” (Opal mIHC, PerkinElmer, Waltham, MA, USA) [30], “in silico multiplexing workflow” [31], “tissue-based cyclic immunofluorescence” (t-Cycif), MultiOmyx (MxIF) and “multi-epitope-ligand cartography” (MELC) technology as well as DNA barcoding-based techniques such as “CO detection by InDEXing” (CODEX, Akoya Biosciences ,Marlborough, MA, USA) and GeoMx^®^ (NanoString, Seattle, WA, USA). IF-based methods are much more effective and faster than the IHC-based methods, given that more than one marker can be stained simultaneously in each staining round [14,31,32,33,34,35]. The Opal mIHC technique is based on sequential staining rounds, and the secondary antibodies are tagged with tyramide signal amplification system (TSA)-conjugated fluorescence molecules. Heat-treated stripping of the tissues in between the staining rounds removes the primary and secondary antibodies but not the TSA-conjugated fluorescence molecules. After multiple staining rounds, the slides can be acquired. There is no limitation in the number of different antibody species but there is a restriction in the number of fluorochromes [30]. Blenman et al. established a workflow for multiplexing that includes multiple staining rounds of the tissue, whole-slide imaging with the tissue cytometer TissueFAXS PLUS (TissueGnostics, Vienna, Austria), dye inactivation by chemical bleaching after each acquisition step, as well as merging the images from all staining rounds and quantitative analysis of the stained markers/cell populations with StrataQuest software (TissueGnostics) [31]. A similar strategy is used by the t-Cycif and the MxIF techniques [32,33]. One big advantage of these chemical bleaching-based methods is that they substantially reduce autofluorescence of the tissue after each acquisition step [36]. However, chemical bleaching-based technologies are still time consuming; for a staining protocol of 30 markers, approximately 1–2 weeks are needed. One main disadvantage of the repeated chemical-based bleaching steps for fluorochrome removal after each staining/imaging round is that the preservation of cell and tissue integrity cannot be guaranteed. Lin et al. reported that after 10 staining rounds, a loss of 2–46% of the cells within various tissue types was observed [31,32,33]. Another technology used for multiplexing is MELC, which is based on fully automated and repeated rounds of multiple marker IF staining, imaging as well as chemical and photobleaching (at the excitation wavelength) of the fluorochromes on a tissue section. The main limitation of the MELC technology is that the photobleaching and imaging step can be only applied to one microscopic field of view [35]. A rather innovative and novel technology able to deal with a very high number of different target antigens is the DNA barcoding-based method CODEX. A cocktail of up to 50 unique oligo-DNA (barcodes) conjugated antibodies specific for the target markers is applied at once on the tissue section. Next, the barcodes are detected by highly specific dye-labeled reporters, which are barcode-complementary oligonucleotides labeled with fluorochromes. Multiple rounds of staining, imaging, and removing of the reporters allow high-dimensional phenotyping [34]. Similar technology is used by GeoMx^®^ (NanoString, Seattle, WA, USA), which is also based on oligonucleotide tags (barcodes) in combination with microscopic imaging to identify a high number of markers (proteins, mRNA, miRNA, etc.) in one hybridization reaction [26]. A summary of the above-mentioned staining methods is provided in Table 1.

The enhanced number of stained markers offered by several multiplexing methods also increases the necessity of appropriate next-generation digital pathology platforms that provide fully automated acquisition of the stained tissue sections as well as computer-assisted/digital high-content phenotypic analysis and high-dimensional data mining.

## 3. Advanced Imaging for Digital Pathology

The first step in a tissue cytometry/next-generation digital pathology workflow includes whole slide scanning or at least acquisition of a region of interest of the stained slide. The second and even more important step comes with the subsequent computer-assisted quantitative image analysis. Next-generation digital pathology technology aims to guide the workflow away from visual observation with a standard microscope and subjective estimations, which are funneled into scoring schemes describing marker expression with “+/++/+++”, to a fully automated computerized platform for the detection and numerical quantification of stained markers in defined cell subpopulations in relation to specific histological structures. Not only are these platforms providing a fast analysis of markers, but they also seek accurate, unbiased, reproducible, and standardized results. These platforms are already well integrated and used in various fields of research [37,38]. Additionally, in 2017 the FDA approved the first next-generation digital pathology program (Philips IntelliSite; PIPS) as a clinical digital diagnostics tool in routine diagnosis [39].

Several whole slide imaging platforms (with or without image analysis software) are commercially available in various configurations (e.g., TissueGnostics, Akoya Biosciences, Leica Biosystems, Hamamatsu, Zeiss, 3DHistech, PerkinElmer, Roche, Philips). As the name already indicates, these scanners are able to acquire whole slides instead of only individual captures of fields of view, and thereby provide complete composite digitized images of slides in high resolution. The technology used is image acquisition by either tile scanning or line scanning with a follow-up stitching of the images [14,40]. Depending on the specific next-generation digital pathology platform configuration, these scanners are able to perform whole slide imaging in different imaging modes such as brightfield, widefield fluorescence, confocal, structured illumination, multiplexing, and/or multispectral. The hardware components are usually the following: microscopy stand (upright, inverted) or boxed system without a phototube, cameras (color and/or monochrome), light sources for fluorescence and/or brightfield mode, multiple filter sets for multicolor fluorescence imaging (may include single-, dual-, and/or multi-band filters), high-quality objective lenses for acquisition with different magnifications (1× to 100×), motorized slide scanning stage or high-throughput slide loading systems. Some platforms offer objective auto-oiling for high magnifications and/or provide a slide bar-code reader for higher efficiency. A powerful computer workstation and high-resolution computer monitors for the viewing of the digitized slide as well as for the potential follow-up image analysis [37,41] are mandatory. For controlling all the individual components, for digitized slide viewing and data management, the platforms also include highly functional slide imaging software [40]. In some instances, the platforms also offer or are equipped with image analysis solutions for quantitative analysis. Such quantification is not stoichiometric, and hence does not provide chemical concentration of markers, but is rather based on comparison with negative controls, which is referred to as cytometric.

The basic Theory of Scales of Measurements defines four different types of scales—nominal, ordinal, interval, and ratio [42], the first one being referred to as qualitative, and the other three being accepted as quantitative. In all three scales, systematic, observer-independent measurements of well-defined attributes of objects can be performed, resulting in numerical values that allow for comparison of the objects under investigation as well as statistical evaluation of the assigned attributes.

Most slide scanners today provide area and distance measurements in metric values, whose measurements fall into the ratio type of scales. The amount of any given molecular marker expressed in certain cells, however, is usually determined as a relational value (e.g., mean relative fluorescence or optical density in brightfield microscopy) rather than an absolute value (e.g., µmol or nanogram). Hence, such measurements belong to the interval type of scale. Such measurements do permit comparative measurements of more and less, but it is not possible to draw conclusions by building the ratio between two values. If a cell or cell population expresses a certain molecule at a mean relative fluorescence of 7000 and another cell or cell population exhibits a value of 14,000, in the scope of a cytometric measurement it is safe to state that “the second cell/population contains more of that molecule than the first” and that “the mean relative fluorescence increases from 7000 to 14,000” in a comparison of these two entities, but it cannot be concluded that the amount of molecules doubles. This is similar to our daily temperature readings in Celsius or Fahrenheit: 30 °C is not “twice as hot as 15 °C”. Cytometric measurements belong to the interval scale and are thus to be considered quantitative.

**Figure 1:**
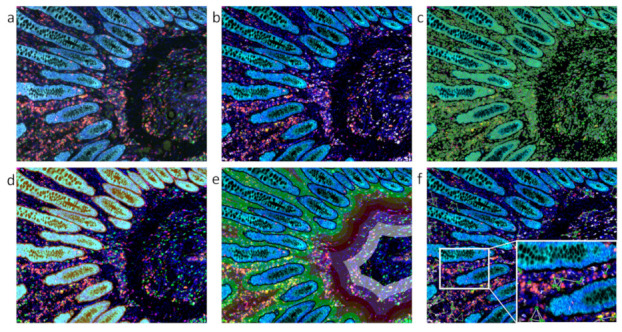
A representative example of high-dimensional automated tissue cytometry shown on a colon sample stained for seven markers. (**a**) Original multicolor immunofluorescence image data set acquired by a multispectral imaging technology. Nuclei stained by 4′,6-diamidino-2-phenylindole (DAPI) in blue; immune markers/immune checkpoint markers CD4 in green/PD-L1 in yellow/PD1 in red/CD68 in pink/CD8 in orange; pan-cytokeratin marker in turquoise. As this raw data image contains overlapping emission signals from the fluorochromes, the colors appear mixed. (**b**) Image with clearly separated fluorescent signals obtained by a mathematical procedure referred to as spectral unmixing. (**c**) Nuclei detection, highlighted by the green contour mask shown in overlay to the original image. (**d**) Metastructure detection of epithelial cells, highlighted in orange overlay. (**e**) Proximity measurements in relation to detected metastructures with various distance zones highlighted by different colors. (**f**) Analysis of spatial connections among and between single cells of a specific cellular phenotype highlighted by a green mask and white connecting lines. The images were provided by and analyzed using TissueGnostics’ image cytometry solution StrataQuest.

Image analysis options can provide unlimited applications depending on the platform and among others may enable basic single cell analysis, dot detection, cellular co-expression as well as subcellular co-localization analysis, meta structure detection, multiplexed high-content phenotyping, proximity measurements, structural tracing (e.g., neurites and/or axons), particle and/or single cell tracking, as well as the analysis of spatial relationships for next-generation digital pathology [41,43]. A representative example of high-dimensional data analysis and the power of these platforms is shown in Figure 1.

## 4. Role of Machine Learning

A big fundamental improvement step in recent years elevating the next-generation digital pathology approach is the integration of artificial intelligence (AI) algorithms for pattern recognition into the image analysis/image cytometry process [44]. Over the past few years, these AI tools have become more robust, and with only minimal user input can be applied to automatically detect objects such as nuclei and specific structures as well as for the classification of various anatomical tissue entities within an entire digitized slide [44,45].

Understanding molecular and cellular interdependencies quickly leads to complex questions, which require the elaboration of extensive algorithms and enormous amounts of computing power to get to an answer. While machine learning has been known to have great potential in this field for many decades, in the recent past it has advanced greatly in its practical use due to the availability of powerful computer technology, in particular parallel computing on multiple CPUs and/or CPU cores as well as due to new software tools, programming languages, and advanced machine learning techniques, which have made the technologies much easier to use without the requirement of advanced theoretical knowledge [44,45]. By engaging state-of-the-art technologies, computer scientists and engineers try to generate models that can provide answers to the complex problems given by nature. Machine learning’s power resides in its robustness in generating customized models designed to solve (very) specific problems. [44]. Machine learning models are generated by learning on examples consisting in observations. Current techniques of machine learning comprise supervised, unsupervised, transfer, federated, and reinforcement learning [45].

In the case of supervised machine learning, the observations are tagged to a class by a human expert, and therefore the model efficiency is strictly related to the quality of the training data set used. An optimal training data set should cover a wide enough range of variability expected in the real-world data, for example a well annotated slide. Failing to do so can increase the possibility of misclassifications. [46].

Unsupervised learning refers to a machine learning method where the algorithm learns from examples without being able to refer to predefined target values or classes (untagged/unlabeled data). The algorithm tries to identify patterns by creating an internal representation of the data and looks for density probabilities (e.g., clustering analysis). This method is suited to search for patterns which are not obvious, or are difficult to identify even for/by the human eye [47].

The transfer machine learning method is characterized by the fact that an already trained algorithm can be used to answer different, but related questions. It means an existing trained model can be adapted/tweaked to solve new tasks without the need to train a new model from scratch [48].

In the federated machine learning method, the algorithm learns from data spread/stored on multiple devices. Federated machine learning is similar to distributed learning, but the focus is on training on heterogeneous data and not on parallelization. No training data information is shared between the devices as part of the learning process [49].

Another machine learning method, enforcement learning, is based on an algorithm that needs to take optimal decisions based on the new data presented and the cumulated experience (knowledge). The learning process is continuous; each decision taken by the algorithm is labeled using a system with rewards and punishments. The aim is to solve the task by maximizing the cumulative positive feedback [50]. Precisely, the step-by-step development in machine learning aims towards a human-like learning, in the sense that humans learn from existing experience even in unrelated sectors and can transfer knowledge to new arising tasks rather than start from the basics, which is, however, still the case in machine learning.

Due to the versatile range of applications of next-generation digital pathology discussed in the following section, these platforms (with or without AI) can be seen as a crucial part of precision medicine by providing a solid and fully automated tool for the gaining of novel information on the pathology of specific diseases, identification of novel predictive and prognostic biomarkers, as well as targets for therapy [37,38,44].

## 5. Current Applications of Next-Generation Digital Pathology

### 5.1. RNA In Situ Hybridization (ISH)

**Figure 2:**
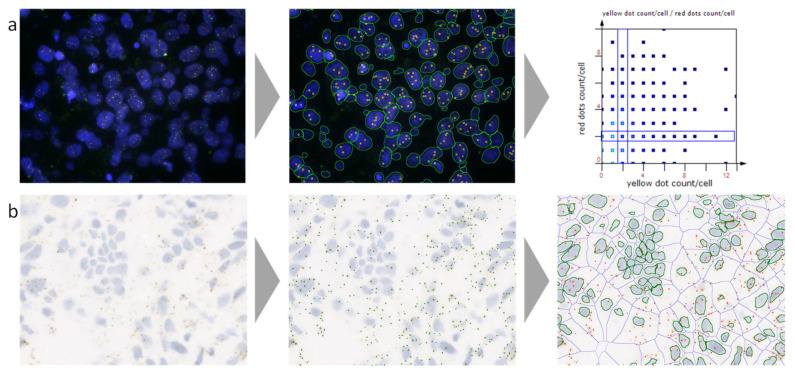
A representative example of automated analysis of fluorescence in situ hybridization (FISH) and RNA in situ hybridization (ISH) stained cells using a next-generation digital pathology platform. (**a**) FISH staining (blue, nuclei stained for 4′,6-diamidino-2-phenylindole (DAPI); red and yellow dots, FISH probes); on the left the original image is shown, in the middle the corresponding analyzed image including cell and dot detection mask, and on the right the analyzed data visualized in a scattergram. (**b**) RNAscope staining (blue, nuclei stained for hematoxylin; brown, RNAscope staining); on the left the original image is shown, in the middle the original image overlaid with the detected dot mask, and on the right the original image overlaid with the nuclei mask, the cellular mask, and the identified dot mask. Both images were provided by and analyzed using TissueGnostics’ image cytometry solution StrataQuest.

In clinical settings, a routinely used method to measure RNA is real-time PCR [51]. However, this grind-and-bind technique is unable to visualize the individual cell signals in their original context, and is prone to becoming contaminated by unintended cell and tissue types and masking the different cellular subpopulations and phenotypes in the heterogenous TME [6,52]. Next-generation sequencing and single-cell sequencing technologies can detect RNA expression at the single cell level, but dissociation from their native setting deprives the data related to their spatial relationship [53]. With the latest developments in RNA ISH, multiple approaches came into play such as non-isotopic fluorescently labeled ISH (fluorescence in situ hybridization—FISH) and biotin or hapten labeled nucleic acid probes (chromogenic in situ hybridization—CISH) to gather spatial data [52,54,55,56,57]. These methods opened a new data dimension, supporting localization and quantitation of target RNA in single cells to detect precise RNA expression in specific cell types [52,58]. However, these techniques only allow a restricted number of labels to be integrated into the probes, leading to reduced sensitivity of expression for most of the genes [52]. Due to a high possibility of cross-hybridization and non-specific binding in complicated tumors, the signal-to-noise ratio is constrained, and extreme technical complication limits the performance of these methods [52,58]. In Figure 2, a representative example of the automated quantitative analysis of FISH and RNA ISH is shown.

RNAscope by Advanced Cell Diagnostics Inc., Hayward, CA (ACD) has presented the most pragmatic method that overcomes these limitations of traditional RNA ISH by a unique probe design and an advanced signal amplification system [52,59]. This technology excels due to its specificity, sensitivity, low turnaround time, and robustness in a wide range of applications across various disciplines including infectious diseases, neuroscience, cell or gene therapy, and single-cell transcriptomic profiling in cancer [52,60,61,62,63,64]. In the TME, RNAscope has prominent advantages such as spatially mapping a cell atlas [65,66], visualizing and characterizing gene signatures and generating the immune landscape, and even identification of novel cell subtypes [67,68], classifying and identifying highly heterogeneous and immunotherapeutic cell types [69,70], and identification and characterization of a gene signature of stem cells [71,72,73] and circulating tumor cells [74,75] as well as analyzing or predicting their response to drug treatments [76,77]. Compared with a one-probe RNA ISH hybridization system, the possibility of nonspecific amplification in RNAscope is considerably low since it implies a double-probe independent hybridization system and improves the sensitivity and the signal-to-noise ratio, allowing better quantification of RNA expression [52,78].

The RNAscope method allows robust detection of mRNA, long non-coding as well as microRNAs [57,79,80,81,82], and multiple gene transcripts generated by alternative splicing [83,84] simultaneously in fresh-fixed, fresh-frozen, and formalin-fixed paraffin-embedded (FFPE) clinical specimens, revealing the full potential of RNA [85]. For example, the expression of a majority of androgen receptor (AR) splice variants other than the full-length AR variant remains unclear in prostate cancer progression. RNAscope has been proposed to be a capable technique for detecting expression and localization of splice variants by designing probes specifically to target distinct splice variants. For example, AR and AR-V7 expression have been detected in FFPE prostate tumors by RNAscope where AR expression was found to be 3-fold higher in primary tumor cells compared with benign glands, while AR-V7 expression was higher in metastatic castration-resistant prostate cancer than in primary prostatic tissues [84].

Emerging new therapeutic strategies broadly target both cellular and non-cellular components of the TME more than ever, by various therapies such as immune checkpoint blockade therapy, dendritic cell vaccination, and antiangiogenic therapy [86]. Detection of RNA targets in the TME that are involved in tumor immunotherapy with the RNAscope assay can facilitate these therapies predominantly. RNAscope applications enable the determination of localization of specific immune cell types (i.e., cytotoxic lymphocytes and regulatory T cells) in the TME [87], spatial relationships between different cell types in the TME [88], and immune activation state and function of tumor-infiltrating immune cells in the TME [89,90]. For example, Monte et al., using RNAscope assay, reported that infiltrating basophils in the TME regulate tumor-promoting Th2 inflammation and reduce survival in pancreatic cancer patients [89]. Besides, this technique is an attractive strategy to determine cell type-specific expression of immune checkpoint markers [91] and differentiate activated CAR+ T cells from endogenous T cells [5]. RNAscope’s aptitude to precisely identify the cellular sources of secreted proteins (e.g., cytokines and chemokines) is a distinct benefit since although the mRNA will always localize in the cells of origin, secreted proteins tend to dilute and diffuse in the intercellular space [67,87,92]. Besides, RNAscope provides valuable information on the differentiation of paracrine and autocrine signaling, which aids in the classification of subtypes of several cancers [93]. A dual gene analysis approach with RNAscope has been utilized for simultaneous detection of CD44+ cells and PD-L1 in head and neck squamous cell carcinoma, which found that CD44+ in the TME induces expression of PD-L1, thus subsequently suppressing T cell-mediated immunity in the TME [94]. The localization and quantification of multi-RNA from several genes simultaneously by RNAscope provide greater time saving and significant results from a single feasible technique. However, rapid mRNA translation and RNA degradation in cells can affect RNAscope applications, and thus BaseScope, a subfield of RNAscope, has been recommended for short RNA targets of 50–300 nucleotides [95]. Instead of using 20 probe pairs, BaseScope utilizes short 1–6 probe pairs to target small regions of RNA more effectively. Thus BaseScope is a successful method to determine the expression and quantification of small nucleolar RNAs (snoRNAs), microRNAs, and the RNAs which have a high potential of degradation and transient expression in the TME [95].

The newest approach of RNAscope, in combination with IHC and called dual RNAscope ISH/IHC, has proven to offer an ideal platform to generate more reliable data that can be used to study gene expression signatures at the RNA and protein level with spatial and single-cell resolution in complex TME [5]. This allows correlation of both RNA and protein expression in a single slide, simultaneously validating antibody specificity [78,96,97,98]. For example, combined detection of HPV RNA by RNAscope and Cdc2 protein expression by IHC has been useful to predict the prognosis of oropharyngeal squamous cell carcinoma patients. Even more, the results conclude that the sensitivity of RNAscope was higher than that of PCR reverse dot hybridization [98]. The automated RNAscope is a significant advancement over manual RNAscope and improves the clinical advantage by allowing more samples to be analyzed in a standardized way simultaneously with less time, less inter-user variability, and less manpower in an observer-independent manner [86]. The method has proven consistent and provides reproducible results in quantifying transcript levels. Overall, the spatial resolution presented by the RNAscope method brings a novel dimension to precise localization of target RNA in single cells and allows localization and quantitation of RNA expression in specific cell types in the TME [86].

### 5.2. Assessment of the Tumor Immune Microenvironment

One of the most promising fields in biomarker and therapy target detection in oncology is dedicated to the exploration of the patient-specific immune contexture in situ with conventional and multiplexing IF and IHC staining techniques in combination with automated quantification [14].

One prominent approach for immune cell assessment within a particular tumor tissue, colorectal cancer (CRC), was developed by the group of Galon et al., where they successfully established a patient stratification strategy based on the detection/identification of T cell populations within the tumor core and the invasive margin named Immunoscore (ratio of the markers CD3 and CD45RO, CD3 and CD8, or CD8 and CD45RO). It is currently undergoing evaluation/implementation as a routine parameter for prognostic and predictive diagnosis in clinics for colon cancer [99,100]. To demonstrate its power the group of Pages et al. conducted a large-scale study, where his group assessed the Immunoscore by using a digital pathology method of a large patient cohort (*n* = 2681 CRC patients), aligned it with clinical pathological data, and thereby was able to show the power of the Immunoscore in the prognosis of survival prediction and treatment response in CRC patients [101]. In order to provide a representative (yet not complete) overview of recent applications, Table 2 shows further examples of studies using conventional and/or multiplexing IF and/or IHC staining techniques in which next-generation digital pathology was the central method for the quantification of various immune cell markers/populations in different cancer types and aligned with clinicopathological parameters.

The examples summarized in Table 2, as well as the example shown in Figure 3 from Desbois et al. [153] show the immense power of the applications of this technique utilizing next-generation digital pathology for the assessment of the immune tumor microenvironment. In order to integrate the Immunoscore or other immune cell screening strategies also into clinical research, such fully automated next-generation digital pathology platforms should be implemented into the process of quantification of the rate of infiltration of various immune cell populations/markers. Ongoing clinical studies are aiming at the integration of such platforms in combination with the staining of a set of immune-related biomarkers including main subpopulation markers and immune checkpoint markers [14].

To sum up, the need to automatically assess immune cell markers in situ, as well as analyzing spatial relationships, and thereby providing a better understanding of various immune cells populations and their interactions, is crucial for the detection of novel predictive and prognostic biomarkers as well as for clinical therapy strategy.

### 5.3. Detection of Blood Vessels

Neoangiogenesis and the resulting vascularization are equally required by the tumor, as in healthy tissues. In both types of tissue, normal and tumor, cell survival and proliferation depend on oxygen and nutrition supply as well as on removal of carbon dioxide and metabolic wastes. In contrast to regulated neoangiogenesis in healthy tissues, tumor angiogenesis is characterized by an uncontrolled, ineffective, often incomplete (and therefore leaky) growth of new blood vessels within the tumor tissue in order to supply the tumor mass with oxygen and nutrition [172]. However, the in situ assessment of the density of blood vessels stained by specific markers such as CD31 or CD34 was shown to correlate with the aggressiveness of the tumor in a variety of tumor types such as CRC, breast cancer, gastric cancer, and small cell and non-small cell lung cancer [173]. Furthermore, specific therapies such as neutralizing antibodies targeting anti-vascular endothelial growth factor are widely used in several cancer types [174]. However, inhibition of vessel growth has only been shown to provide limited or even no long-term improvement for cancer types including hepatocellular carcinoma and CRC [175,176]. However, the use of different non-standardized methods for detection and quantitation of blood vessel density leads to contradicting data in terms of influence on patient survival [177]. Therefore, the unbiased automated quantification of blood vessels could help to identify patient groups that would benefit from anti-angiogenic therapies.

Summarized in Table 3 are studies where next-generation digital pathology was used to detected blood vessels/blood vessel densities. Thereby we want to emphasize that the next-generation digital pathology approach is highly versatile and can be applied to various research needs and questions, not only to single cell detection or dot (RNA ISH) detection but also for the analysis of more complex structures such as blood vessels.

## 6. Conclusions

Within this review, we show several application fields that contribute to next-generation digital pathology, including the analysis of RNA ISH, conventional and/or multiplexed immunophenotyping, and blood vessel detection in the tumor microenvironment. Due to new staining technologies that allow a higher number of markers, one of the new challenges is high-dimensional data mining, which needs to be addressed by next-generation digital pathology platform providers. Several platforms are available on the market tackling different kinds of requests, including slide scanning, management of a large amount of data, follow-up image analysis with integrated AI modules, as well as high-dimensional data mining. Next-generation digital pathology has the potential to elevate research and clinics by providing automated, unbiased, fast, reproducible, and therefore reliable image cytometry.

The integration of data obtained by automated analyses, referring to the levels of DNA, RNA—including non-coding RNA—and proteins, will allow the development of tools for research and diagnostics within the scope of precision medicine, i.e., with a focus on the molecular mechanisms involved in disease formation in individual patients rather than averaged cohorts, in which individual yet decisive details get lost. This is the expanding area of tissue cytometry—and the era of next-generation digital pathology.

## Figures and Tables

**Figure 3:**
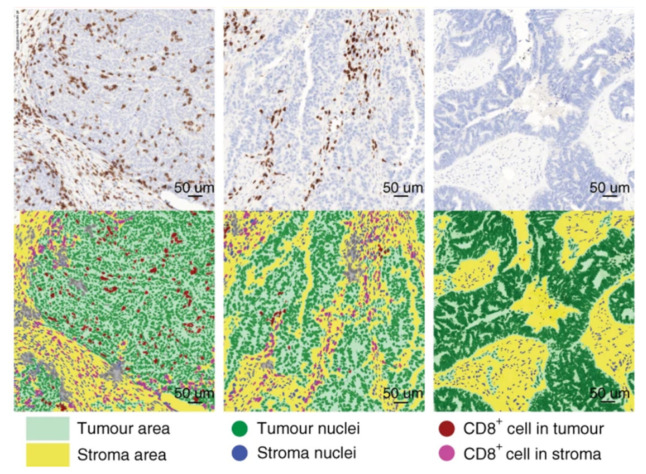
Analysis of the tumor immune microenvironment using next-generation digital pathology. A representative example of the automated detection of CD8+ immune cells within the tumor microenvironment of ovarian cancer by Developer XD (Definiens, Munich, Germany). Figure adapted from Desbois et al., 2020 [153].

**Table 1:** IHC and IF multiplexing techniques.

Method	Process	Advantages	Disadvantages	References
MICSSS (IHC)	Multiple staining rounds; AEC removal AEC with organic solvent-based destaining buffer; imaging	No limitation by the number of different antibody species No company-specific reagents/devices are needed	Time intensive Limited to 10 staining rounds	[29]
SIMPLE (IHC)	Multiple staining rounds; AEC removal with organic solvent-based destaining buffer; imaging	No limitation by the number of different antibody species No company-specific reagents/devices are needed	Time intensive Limited to 5 rounds of staining without loss of tissue antigenicity	[28]
Opal mIHC (IF)	sequential staining with AB tagged with TSA conjungated fluorescence molecules, AB removal by heat-treated antibody stripping; imaging	No limitation by the number of different antibody species up to 7 markers	Time intensive limited by the number of fluorochromes	[30]
In silico multiplexing workflow (IF)	Multiple staining rounds; Dye inactivation by bleaching with alkaline solution + H_2_O_2_; imaging	No limitation by the number of different antibody species No company-specific reagents/devices are needed	Each round of staining may take at least 24 h depending on the antibodies and the tissue	[31]
t-Cycif (IF)	Multiple staining rounds (like MxIF); bleaching by hydrogen peroxide, intense light and high pH; imaging	Background noise decreases with cycle number due to multiple rounds of fluorophore bleaching No limitation by the number of different antibody species No company-specific reagents/devices are needed	Relatively slow (each cycle 6–8 h, most time consuming is the imaging) after 10 cycles, 2–45% loss of cells	[32]
MxIF (IF)	Multiple staining rounds; Alkaline oxidation chemistry was developed that eliminates cyanine-based dye fluorescence within 15 min; imaging	No limitation by the number of different antibody species No company-specific reagents/devices are needed Removal of fluorescence dye within 15 min Up to 60 biomarkers	relatively slow due to scanning times	[33]
MELC (IF)	Multiple automatic staining rounds; during each cycle the sample is incubated with one or more tags and imaged before bleaching by soft multi-wavelength excitation	Automated cycles of fluorescent staining, imaging and photobleaching No limitation by the number of different antibody species	Bleaching/acquisition can be applied only to one field of view Special devices are needed	[35]
CODEX (IF)	Antibodies conjugated to a CODEX barcode; visualized by the binding of highly specific corresponding dye-labeled CODEX reporter	No limitation by the number of different antibody species -> no secondary antibodies Fast, each round of extension and bleaching (10 min) Up to 35 rounds with 3 markers	Special devices and reagents are needed	[34]
NanoString (IF)	Antibodies conjugated to a barcode; visualized by the binding of highly specific corresponding dye-labeled reporter	Up to 40 markers No autofluorescence and spectral overlap	Limited number of regions of interest Special devices and reagents are needed	[26]

AB, antibody; AEC, 3-amino-9-ethylcarbazole; CODEX, co detection by indexing; IF, immunofluorescence; IHC, immunohistochemistry; MELC, multi-epitope-ligand cartography; MICSSS, multiplexed immunohistochemical consecutive staining on a single slide; SIMPLE, sequential immunoperoxidase labelling and erasing method; TSA, tyramide signal amplification system; t-Cycif, tissue-based cyclic immunofluorescence.

**Table 2:** Studies using next-generation digital pathology for the assessment of the tumor immune microenvironment.

Cancer Type	Markers	Scanner/Microscope	Quantification System	Reference
Breast cancer	CD4, CD8, Foxp3	Olympus BX51 (Olympus, Tokyo, Japan)	UTHSCSA Image Tool (University of Texas Health Science Center at San Antonio, San Antonio, TX, USA)	[102]
Breast cancer	CD4, CD8, CD3, CD20, FOXP3, CD68	Leica SCN400 F (Leica Biosystems Inc., Richmond, IL, USA)	ImageJ software (NIH, Bethesda, MD, USA)	[103]
Breast cancer	PD-L1	Aperio AT2 Scanner (Leica Biosystems Inc., Richmond, IL, USA)	QuPath (University of Edinburgh, Edinburgh, UK)	[104]
Breast cancer	CD8	ScanScope XT (Aperio Technologies, Vista, CA, USA)	HALO (Indica Labs, Albuquerque, NM, USA)	[105]
Breast cancer	CD3, CD20, Foxp3	NanoZoomer (Hamamatsu Photonics, Hamamatsu City, Japan); Panoramic 250 Flash (3Dhistech, Budapest, Hungary)	ImageJ software (NIH, Bethesda, MD, USA)	[106]
Breast cancer	CD3, CD8, CD20	NanoZoomer (Hamamatsu Photonics, Hamamatsu City, Japan)	ImageJ software (NIH, Bethesda, MD, USA)	[107]
Breast cancer	CD4, CD68, CD8, FOXP3, PD-L1	Vectra 3 (PerkinElmer, Waltham, MA, USA)	inForm (PerkinElmer, Waltham, MA, USA)	[108]
Breast cancer	CD4, CD8, FOXP3, CD20, CD33, PD-1	Vectra 3 (Akoya Biosciences, Marlborough, MA, USA)	inForm (Akoya, Marlborough, MA, USA)	[109]
CRC	CD3, CD8	n.s.	Developer XD (Definiens, Munich, Germany)	[101]
CRC	CD3, CD8	VENTANA iScan HT (Roche, Basel, Switzerland)	automated image analysis algorithm	[110]
CRC	CD8	Aperio XT Scanner (Leica Biosystems Inc., Richmond, IL, USA)	HALO (Indica Labs, Albuquerque, NM, USA)	[105]
CRC	CD3, CD8	Zeiss Axio Scan.Z1 (Zeiss, Jena, Germany)	HALO (Indica Labs, Albuquerque, NM, USA)	[111]
CRC	CD3, CD4, CD8, CD45RO, FOXP3, Granzyme B, CD15, CD20, S100, CD68, IL17, CD57,	microscope (Leica, Wetzlar, Germany)	TMAJ software (Johns Hopkins University, Baltimore, MD, USA)	[112]
CRC	FoxP3, CD8, CD66b, CD20, CD68	Vectra 3 (PerkinElmer, Waltham, MA, USA)	inForm (PerkinElmer, Waltham, MA, USA)	[113]
CRC	SOX2, CD3, CD8 FoxP3, ALDH1, CD44v6, CD133, Lgr5, PD-L1	Aperio XT Scanner (Leica Biosystems Inc., Richmond, IL, USA)	Aperio Imagescope (Leica Biosystems Inc., Richmond, IL, USA)	[114]
CRC	CD8, CD11c, PD-L1	Pannoramic MIDI II (3Dhistech, Budapest, Hungary)	StrataQuest (TissueGnostics, Vienna, Austria)	[115]
CRC	CD8, CD4, CD20, Foxp3, CD45RO,	Vectra Polaris (PerkinElmer, Waltham, MA, USA)	inForm (PerkinElmer, Waltham, MA, USA)	[116]
CRC, CRCLM	CD20, CD3, Ki67, CD27	TissueFAXS PLUS (TissueGnostics, Vienna, Austria)	HistoQuest, TissueQuest (TissueGnostics, Vienna, Austria)	[117]
CRC, CRCLM	CD8, Foxp3, CD68, CD31	ScanScope (Aperio Technologies, Vista, CA, USA)	GENIE (Aperio Technologies, Vista, CA, USA)	[99]
CRCLM	CD45, CD20	TissueFAXS PLUS (TissueGnostics, Vienna, Austria)	HistoQuest, TissueQuest (TissueGnostics, Vienna, Austria)	[118]
CRCLM	CD3, CD4, CD8, CD20, CD68	NanoZoomer (Hamamatsu Photonics, Hamamatsu City, Japan)	Visilog 9.0 software (Noesis, Saclay, France)	[119]
CRCLM	CD3, CD8, CD45RO, Foxp3, CD20	NanoZoomer (Hamamatsu Photonics, Hamamatsu City, Japan)	Developer XD (Definiens, Munich, Germany)	[120]
Gastric cancer	PD-L1, CD8	digital slide scanner (3Dhistech, Budapest, Hungary); TissueFAXS (TissueGnostics, Vienna, Austria)	QuantCenter (3Dhistech, Budapest, Hungary); TissueQuest (TissueGnostics, Vienna, Austria)	[121]
Gastric cancer	CD68, CD163, CD3, MPO, Foxp3.	ScanScope CS (Aperio Technologies, Vista, CA, USA)	ImageScope (Aperio Technologies, Vista, CA, USA)	[122]
Gastric cancer	CD3, CD4, CD8, PD-1	ScanScope CS2 (Aperio Technologies, Vista, CA, USA)	ImageScope (Aperio Technologies, Vista, CA, USA)	[122]
Gastric cancer	CD8, FoxP3	ScanScope XT (Aperio Technologies, Vista, CA, USA)	image analysis system—ScanScope XT (Aperio Technologies, Vista, CA, USA)	[123]
Gastric cancer	CD8, Foxp3	n.s.	Aperio image analysis system (Leica Biosystems Inc., Richmond, IL, USA)	[124]
Gastric cancer	CD8, Foxp3, CD3, CD56	Vectra Multispectral Imaging System version 2 (PerkinElmer, Waltham, MA, USA)	inForm (PerkinElmer, Waltham, MA, USA)	[125]
Gastric and esophageal cancer	CD3, CD8	n.s.	HALO (Indica Labs, Albuquerque, NM, USA	[126]
Gastric cancer and metastasis	PD-L1	n.s.	Aperio Imagescope IHC Membrane Image Analysis software (Aperio Technologies, Vista, CA, USA)	[127]
HCC	CD3, CD8	n.s.	ImagePro Plus (Media Cybernetics, Rockville, MD, USA)	[128]
HCC	CD3, CD8	Nikon E600 (Nikon, Tokyo, Japan);	ImageJ software (NIH, Bethesda, MD, USA)	[129]
HCC	CD3, CD15, CD20, CD23, CD68, Foxp3, LTß	Ariol SL-50 (Applied Imaging)	Image analysis system (Applied Imaging)	[4]
HCC	CD3, CD8, PD-1, TIM3	Vectra 3 (PerkinElmer, Waltham, MA, USA)	inForm (PerkinElmer, Waltham, MA, USA)	[130]
HCC	CD3, CD4, CD8, CD20, CD27, CD40, CD38, CD56, CD68, CD138, S100, Granzyme B, Ki67	Mantra (PerkinElmer, Waltham, MA, USA)	ImagePro Plus (Media Cybernetics, Rockville, MD, USA)	[131]
HCC	CD3, CD8, CD45RO,	n.s.	ImagePro Plus (Media Cybernetics, Rockville, MD, USA)	[132]
HCC	FoxP3, CD4, CD8, CD34	Olympus BX51 (Olympus, Tokyo, Japan)	ImagePro Plus (Media Cybernetics, Rockville, MD, USA)	[133]
HNSCC	FOXP3, CD8	n.s.	Visiopharm image analysis software (Visiopharm, Copenhagen, Denmark)	[134]
HNSCC	CD3, CD8	Aperio AT2 scanner (Leica Biosystems Inc., Richmond, IL, USA)	StrataQuest (TissueGnostics, Vienna, Austria)	[135]
Melanoma	PD-L1	Philips Ultra Fast Scanner 300 (Philips, Amsterdam, Netherlands)	HALO (Indica Labs, Albuquerque, NM, USA	[136]
Melanoma	CD20	TissueFAXS (TissueGnostics, Vienna, Austria)	HistoQuest (TissueGnostics, Vienna, Austria)	[137]
Melanoma	CD3, CD8, CD68, SOX10, Ki67	Mantra (PerkinElmer, Waltham, MA, USA)	inForm (PerkinElmer, Waltham, MA, USA)	[138]
Melanoma	CD19, CD20, CD27, CD38, CD138, CD5, CD8, Foxp3, CD4, CD69, CD103, CD45RO, CXCL13, CD21, CD23, Bcl6	Vectra Multispectral Imaging System version 2 (PerkinElmer, Waltham, MA, USA)	inForm (PerkinElmer, Waltham, MA, USA)	[139]
NSCLC	CD8, PD-1	Philips Ultra Fast Scanner 300 (Philips, Amsterdam, Netherlands)	HALO (Indica Labs, Albuquerque, NM, USA	[140]
NSCLC	CD8	NanoZoomer (Hamamatsu Photonics, Hamamatsu City, Japan)	Calopix software (TRIBVN Healthcare, Paris, France)	[141]
NSCLC	PD-L1, TIM, CD3, CD4, CD8, CD57, granzyme B, CD45RO, PD-1, FOXP3	Aperio AT scanner (Leica Biosystems Inc., Richmond, IL, USA)	Aperio GENIE (Leica Biosystems Inc., Richmond, IL, USA)	[142]
NSCLC	CD8, CD4, FOXP3, CD163, CCL17, IL-13	Vectra Automated Quantitative Pathology Imaging System (PerkinElmer, Waltham, MA, USA)		[143]
NSCLC	CD3, CD4, CD8, CD57, granzyme B, CD45RO, PD-1, FOXP3, CD68	Aperio AT scanner (Leica Biosystems Inc., Richmond, IL, USA)	Aperio GENIE (Leica Biosystems Inc., Richmond, IL, USA)	[144]
NSCLC	CD4, CD20, CD8, Foxp3	NanoZoomer (Hamamatsu Photonics, Hamamatsu City, Japan)	Tissue Studio (Definiens, Munich, Germany)	[145]
NSCLC	CD68, CD163, PD-L1,	Mantra (PerkinElmer, Waltham, MA, USA)	inForm (PerkinElmer, Waltham, MA, USA)	[146]
NSCLC	CD8, CD4, Foxp3, CD68	Vectra Multispectral Imaging System (PerkinElmer, Waltham, MA, USA)	inForm (PerkinElmer, Waltham, MA, USA)	[147]
NSCLC	CD3, CD8, Foxp3	ScanScope CS (Aperio Technologies, Vista, CA, USA)	GENIE (Aperio Technologies, Vista, CA, USA)	[148]
NSCLC	CD8, PD-L1	Aperio AT scanner (Leica Biosystems Inc., Richmond, IL, USA)	Developer XD (Definiens, Munich, Germany)	[149]
pulmonary squamous cell carcinoma	CD8, PD-1	ScanScope (Aperio Technologies, Vista CA, USA)	ImageScope (Aperio Technologies, Vista, CA, USA)	[150]
pulmonary squamous cell carcinoma	CD20, CD21, CD23, PNAD, DC-LAMP	Vectra 3 (PerkinElmer, Waltham, MA, USA)	inForm (PerkinElmer, Waltham, MA, USA)	[151]
Oral squamous cell cancer	CD3, CD8, FoxP3, CD163, PD-L1	Vectra (PerkinElmer, Waltham, MA, USA)	inForm (PerkinElmer, Waltham, MA, USA)	[152]
Ovarian cancer	CD8, MHC I, FAP ISH	Panoramic 250 (3Dhistech, Budapest, Hungary),	Developer XD (Definiens, Munich, Germany)	[153]
Ovarian cancer	CD8	TissueFAXS (TissueGnostics, Vienna, Austria)	HistoQuest (TissueGnostics, Vienna, Austria)	[154]
Ovarian cancer	CD8, CD45RO, CD68	Panoramic Flash (3Dhistech, Budapest, Hungary)	Tissue Studio (Definiens, Munich, Germany)	[155]
Ovarian cancer	CD4, CD8, CD20	Aperio scanner (Leica Biosystems Inc., Richmond, IL, USA)	ImageScope (Aperio Technologies, Vista, CA, USA)	[156]
Ovarian cancer	CD8	Vectra (PerkinElmer, Waltham, MA, USA)	inForm (PerkinElmer, Waltham, MA, USA)	[157]
Ovarian cancer	CD8, CD103	TissueFAXS (TissueGnostics, Vienna Austria)	Fiji, Image J software (NIH, Bethesda, MD, USA)	[158]
Ovarian cancer	CD3, CD4, CD8	n.s.	CD3 Quantifier (VM Scope, Berlin, Germany)	[159]
Pancreatic cancer	CD3, CD8, CD4, Foxp3, CK8	Vectra Multispectral Imaging System version 2 (PerkinElmer, Waltham, MA, USA)	Nuance Image Analysis software; inForm (PerkinElmer, Waltham, MA, USA)	[160]
Pancreatic cancer	DC-LAMP, FoxP3, CD68, CD3, CD8, CD4, CD20	Panoramic Flash (3Dhistech, Budapest, Hungary)	ImageJ software (NIH, Bethesda, MD, USA)	[161]
Pancreatic cancer	CD20, CD8, PD1	dotSlide (Olympus, Tokyo, Japan)	ad hoc software	[162]
Pancreatic cancer	CD8	NanoZoomer (Hamamatsu Photonics, Hamamatsu City, Japan)	HALO (Indica Labs, Albuquerque, NM, USA	[163]
Pancreatic cancer	CD8, PD-L1, CD44, CD133	TissueFAXS (TissueGnostics, Vienna, Austria)	TissueQuest (TissueGnostics, Vienna, Austria)	[164]
Pancreatic cancer	CD3	NanoZoomer (Hamamatsu Photonics, Hamamatsu City, Japan)	Tissue Studio (Definiens, Munich, Germany)	[165]
Pancreatic cancer	CD3, CD8, CD20, CD66b	n.s.	ImageJ software (NIH, Bethesda, MD, USA)	[166]
Pancreatic cancer	CD3, CD8	Aperio AT scanner (Leica Biosystems Inc., Richmond, IL, USA)	ImageJ software (NIH, Bethesda, MD, USA)	[167]
Prostate cancer	CD3, CD8, CD20, CD56, CD68, Foxp3	ScanScope XT(Aperio Technologies, Vista, CA, USA)	ImageScope (Aperio Technologies, Vista, CA, USA)	[168]
Prostate cancer	CD20	ScanScope XT (Aperio Technologies, Vista, CA, USA)	ImageScope (Aperio Technologies, Vista, CA, USA)	[169]
Prostate cancer	CD3, CD8, Foxp3	NanoZoomer (Hamamatsu Photonics, Hamamatsu City, Japan)	Aperio Digital Pathology software (Leica Biosystems Inc., Richmond IL, USA)	[170]
Clear cell renal cell carcinoma	CD8, PD-1, LAG-3, PD-L1, PD-L2	NanoZoomer (Hamamatsu Photonics, Hamamatsu City, Japan)	Calopix software (TRIBVN Healthcare, Paris, France)	[171]

CRC, colorectal cancer; CRCLM, colorectal cancer metastasis in the liver; HCC, hepatocellular carcinoma; HNSCC, head and neck squamous cell carcinoma; NSCLC, non-small cell lung cancer; n.s., not specified.

**Table 3:** Studies using next-generation digital pathology for the quantification of blood vessels.

Cancer Type	Markers	Scanner/Microscope	Quantification System	References
Breast cancer	CD34	Olympus BX41 (Olympus, Tokyo, Japan)	Cell D software (Olympus, Tokyo, Japan)	[178]
Breast cancer	CD34	NanoZoomer (Hamamatsu Photonics, Hamamatsu City, Japan)	Slidepath Image Analysis system (Leica Biosystems Inc., Richmond, IL, USA)	[179]
Breast cancer	CD34	TissueFAXS (TissueGnostics, Vienna, Austria)	HistoQuest (TissueGnostics, Vienna, Austria)	[180]
Breast cancer metastasis	CD31	Panoramic 250 (3Dhistech, Budapest, Hungary)	Visiopharm image analysis software (Visiopharm, Copenhagen, Denmark)	[181]
CRC	CD31	Mirax slide scanner system (3Dhistech, Budapest, Hungary)	Image J software (NIH, Bethesda, MD, USA)	[182]
CRC	CD31	TissueFAXS (TissueGnostics, Vienna, Austria)	StrataQuest (TissueGnostics, Vienna, Austria)	[183]
ESCC	CD31	TissueFAXS (TissueGnostics, Vienna, Austria)	HistoQuest, TissueQuest (TissueGnostics, Vienna, Austria)	[184]
Human tumor	CD31, CD34	Aperio (Leica Biosystems Inc., Richmond, IL, USA)	Fiji, Image J software (NIH, Bethesda, MD, USA)	[185]
Melanoma	CD31	Aperio CS Scanner (Leica Biosystems Inc., Richmond, IL, USA)	Aperio image analysis system (Leica Biosystems Inc., Richmond, IL, USA)	[186]
Pancreatic cancer	CD31	n.s.	The Ariol™ image analysis system (Genetix, New Milton, England)	[187]
Renal cancer	CD34	Zeiss Axio Scan.Z1 (Zeiss, Jena, Germany)	Developer XD, Tissue Studio (Definiens, Munich, Germany)	[188]
Rectal cancer	CD34	ScanScope CS (Aperio Technologies, Vista, CA, USA)	ImageScope (Aperio Technologies, Vista, CA, USA)	[189]
Tongue cancer	PNAd	ScanScope T3 (Aperio Technologies, Vista, CA, USA)	Image J software (NIH, Bethesda, MD, USA)	[190]

CRC, colorectal cancer; n.s., not specified; ESCC, esophageal squamous cell carcinoma.
